# When to initiate early palliative care? Challenges faced by healthcare providers

**DOI:** 10.3389/fmed.2023.1220370

**Published:** 2023-10-02

**Authors:** Joel Vieira Vitorino, Beatriz Veiga Duarte, Carlos Laranjeira

**Affiliations:** ^1^School of Health Sciences, Polytechnic of Leiria, Morro do Lena, Alto do Vieiro, Leiria, Portugal; ^2^Palliative Care Unit, Portuguese Institute of Oncology of Coimbra, Coimbra, Portugal; ^3^Centre for Innovative Care and Health Technology (ciTechCare), Polytechnic of Leiria, Leiria, Portugal; ^4^Comprehensive Health Research Centre (CHRC), University of Évora, Évora, Portugal

**Keywords:** palliative care, healthcare providers, assessment, patient-centered care, referral, resources, education, communication

## Introduction

Palliative Care (PC) can begin when a serious illness is diagnosed and continue during the entire continuum of care (1). According to the World Health Organization, PC “is a crucial part of integrated, people-centered health services. Relieving serious health-related suffering, be it physical, psychological, social, or spiritual, is a global ethical responsibility. Thus, whether the cause of suffering is cardiovascular disease, cancer, major organ failure, drug-resistant tuberculosis, severe burns, end-stage chronic illness, acute trauma, extreme birth prematurity, or extreme frailty of old age, palliative care may be needed and has to be available at all levels of care” [(2); p. 1].

Of the 234 nations in the globe, roughly 136 offer PC services and resources. While Europe, Australia, Canada, and the United States have the highest levels of PC integration, many regions of Africa and several regions of Asia and South America lack basic amenities (3). There are several aspects that contribute to the development of PC integration in health care systems: economic resources, cultural and religious aspects, education and training of health care teams (3–5).

The available evidence indicates an increasing need for PC (6–8). This represents a challenge in the definition of health policies (9–11), in the organization of health services and responses (12, 13), but also for health professionals (11, 14).

Recently, the COVID-19 pandemic resulted in many critically ill and dying patients requiring expert management of symptoms “such as dyspnea, pain, and delirium, as well as serious illness communication, including conversations about care goals and end-of-life issues” [(15); p. e22]. Given the increasing uncertainty of the disease's trajectory, high-quality PC must be offered and affordable for all people. However, COVID-19 did complicate matters. When patients deteriorated rapidly, time was of the essence, health workers were overburdened, seclusion was mandatory, and relatives were instructed not to touch or even be in the same room as loved ones (16). The pandemic is therefore considered to have significantly increased emerging PC needs. Likewise, the pandemic slowed and, in some instances, reversed advancements made in PC development (17, 18).

The literature has supported the idea that early referral to PC translates into increased quality of life (19, 20). Therefore, identifying the appropriate time to initiate the palliative approach to the patient is a challenge for formal as well as informal caregivers.

We aim to identify areas of assessment, auxiliary tools, as well as possible paradigm shifts toward a socio-ecological approach to the person with palliative needs, in order to provide differentiated PC adjusted to the person's condition as early as possible.

## Benefits and implications of early palliative care

Several studies report that few people receive PC in their last year of life (21–23). However, earlier PC has been shown to improve quality of life and survival (20, 22, 24, 25). PC has several benefits, including improved quality of life and mood (25), decreased need for therapy at the end of life, and decreased healthcare costs (26). According to Mittmann et al. (22), early identification leads to an increase in access to PC services.

Timely PC is a systematic approach that identifies patients with high supportive care requirements and refers them to specialized PC as soon as possible based on defined referral criteria. According to Hui et al. (27), it needs four elements: routine assessment of patients' needs for supportive care; institution-specific consensual referral criteria; a system to initiate referrals when patients meet requirements; and availability of outpatient PC resources to provide individualized and timely patient-centered care, with the goal of improving patient and caregiver outcomes. That at least some aspects of PC begin earlier, while the patient is still able to communicate effectively and participate as fully as possible in their medical treatment, is crucial to patient-centered care (28). This may also minimize the burden of replacing decision-making by the patient's family or caregiver, thereby decreasing recurrent distress emotions and avoiding more complicated grieving in the future (28).

To provide effective and efficient patient-centered care at the palliative stage, we must attend to the complexity of care and the areas of specialized interventions (29), by clearly defining the core competencies of the multidisciplinary health team members, and prioritizing a holistic and interactive approach (30). Involvement of care teams reduces hospitalization rates for PC patients and enables them to spend more time at home (30), honoring patients' wish to be cared for at home (31).

When contemplating the start of PC, it is crucial to differentiate between the use of a palliative strategy to treatment (primary PC) and referral to specialist PC consulting services. Primary PC is appropriate at all stages of illness and can be delivered by any healthcare provider (21, 28). This palliative approach to care may be included in the care of every patient following diagnosis, as part of a personalized treatment plan. It provides patients, their relatives, and carers greater control, while also improving quality of life and wellbeing. It may include emotional, social, and spiritual components of care, in addition to physical components of care. Healthcare providers may collaborate with patients and their families to respect them as persons and honor their healthcare treatment choices by aligning care with the patient's goals and principles of PC (1, 28). In contrast, specialized consultation by a PC professional where the major focus of the consultation is comprehensive therapy, including advanced symptom management, psychological, social, and spiritual support, and dignity-preserving care. Specialist PC services are often required when symptoms—whether physical, psychological, or multifactorial—are refractory or difficult, and frequently include interdisciplinary therapy with the goal of fostering quality of life and preserving meaning in existence (28).

There is an urgent need to ensure quality and equality of treatment for all PC patients, from those who require a PC approach to those who require expert intense PC (29). Attention need be given to the areas of care requiring evaluation, the strategies to be implemented, the evaluation instruments that can be used, and the consequences resulting from this approach.

Current health-care models frequently rely on referral-based PC, which can lead to uneven access to treatments or patients receiving PC near the end of their illness progression (32). Early PC has been identified as one option for extending access to PC services for patients suffering from illnesses with an unpredictable course, such as organ failure (33). Early PC is a proactive technique for developing treatment goals, controlling symptoms, and improving quality of life in patients with any life-limiting chronic condition, thereby expanding the scope of traditional PC services (34). Clearly defining the early PC approach so that health professionals and patients understand what early PC is and what role it can play, as well as its potential benefits, could assist in overcoming patient- and family-related barriers rooted in the traditional portrayal of PC. Barriers include misinformation, reluctance to accept referral, or the belief that PC is synonymous with terminal care. Similarly, physicians are concerned that referring patients to PC may cause patients and their families to lose hope and experience suffering (35). Teaching physicians how to deliver “bad news” may aid in their ability to deal with the emotional concerns associated with sending patients to specialist PC (35).

Tuca et al. (36) propose an early PC model that includes a multidimensional assessment, allowing clinicians to classify patients as having low, medium, or high palliative complexity, based on the requirement for basic or specialist PC: “(a) Low complexity—capacity and training of a non-specialized PC team is sufficient; referral to a specialized team is not indicated; (b) Medium complexity—care requires more than capacity and training of a non-specialized PC team; a shared care with a specialized PC team is indicated; (c) High complexity—care entails far more than capacity and training of a non-specialized PC team; intensive shared care with specialized PC is required” (p. 242).

PC complexity is defined “as a clinical condition based on the interaction of emerging clinical characteristics according to a multidimensional evaluation, which confers a special tendency to clinical instability, uncertainty in the outcome of health care intervention, and the subsequent need to intensify specialized palliative support measures” [(36); p. 242].

The measurement of days for referral to specialist PC does not always imply that the team is not providing enough care based on the patient's needs (37). The number of persistent symptoms noted in each patient is a significant signal that specialized treatment may be required, since PC professionals may have better expertise treating refractory and chronic symptoms (37).

Support for a PC team, on the other hand, is often delayed or occurs when there is an excess of symptoms and functional dependency. The use of tools can be critical in the early identification of patients with palliative requirements, and they should ideally be accurate, dependable, low-cost, and smoothly integrated into the current workflow (38). Their usage may assist in indicating the appropriate palliative strategy and understanding the demands of the patient with advanced chronic illness. As demonstrated in Table 1, there is a series of instruments that guide the palliative approach via the assessment of prognosis and/or palliative requirements.

**Table 1:** Measurement tools used to assess palliative care needs of patients.

**Tool/instrument**	**Purpose**
Palliative necessities CCOMS-ICO (NECPAL)	Identify patients with advanced chronic disease who require palliative care, namely in general health services (39)
Supportive and palliative care indicators tool (SPICT)	Identify people in a situation of serious and irreversible disease. Allows assessment of the need for palliative care and its planning (40)
Diagnostic instrument of complexity in palliative care (IDC-Pal)	Diagnose and stratify complexity in patients who have palliative care needs (41)
Karnofsky performance status scale	Assess the functional status of cancer patients to determine the type of treatment (42)
ESAS—Edmonton symptom assessment scale	Rates symptom severity and monitor their evolution in patients seen by palliative care in different care settings (43)
PPS—Palliative performance scale	Establish prognosis by assessing functional status (not the disease in question). Applicable in any disease situation (44)
Gold standards framework prognostic indicator guidance (GSF-PIG)	Support early recognition of patients approaching the end of life and promote person-centered care (45)
Integrated palliative care outcome scale (IPOS)	Brief tool for global measurement of perceptions and holistic assessment of the symptoms and other concerns the patient might have (46)
“Surprise question” (SQ1 - Would you be surprised if this patient died within the next 12 months?)	The original purpose of SQ1 is to identify high-risk patients who might benefit from palliative care services (47). If SQ1 is answered with “no” an additional question (SQ2) should be asked in order to more accurately predict deterioration and death (48, 49)
“Double Surprise question” (SQ2—Would I be surprised if this patient is still alive after 12 months?)		
Holistic common assessment		Assess patient needs in palliative care, including aspects like cultural background, mental ability, preferences and priorities (50)

Despite efforts, there is still no consensus on when the palliative trajectory begins, and early integration of PC in clinical practice is still dependent on overcoming the numerous barriers associated with the disease, health professionals, and service organization (37).

## Integrative approach: final remarks

Referral to PC tends to consider clinical aspects, such as the benefit of therapeutic intervention, as a dichotomous alternative to palliative intervention, considering them differentiated, separate approaches rather than attempting their integration in a holistic and complementary way.

Scientific knowledge suggests one should consider the palliative approach in an integrated manner, as a multidimensional and interdisciplinary intervention that views the person holistically. The fragmentation of health by professional area or by isolated clinical gain limits the potential for interventions addressing ecological and social aspects of health. Socio-ecological models have been used to contextualize the effect of many environmental variables on vulnerable people's lives. The person is at the heart of and immersed in a variety of environmental systems, ranging from proximate settings like the family to bigger contexts like culture (51). While PC has long acknowledged physical, psychological, social, and spiritual comprehensive worlds, we must go further. To comprehensively and effectively investigate and answer patients' requests, we must consider “pre-existing and cumulative complexity, the dynamic aspects of complexity, invisible complexity, service/system-level difficulties, and societal repercussions” [(52); p. 1078].

Illness as a condition of vulnerability, and therefore attending to a patient's specific needs, based on a client-centered care perspective, including all their dimensions (physical, psychosocial, and spiritual) is an ethical and moral imperative of health professionals. Thus, more than defining a chronological time to begin the palliative approach, it is important to consider the individual's time in meeting their real needs, incorporating PC in the practice of professionals, resorting to the expertise of differentiated professionals whenever they can contribute to the person's fulfillment in each moment of their experience of the processes of illness.

## Author contributions

All authors listed have made a substantial, direct, and intellectual contribution to the work and approved it for publication.
